# Diffusion-Weighted Images Superresolution Using High-Order SVD

**DOI:** 10.1155/2016/3647202

**Published:** 2016-08-18

**Authors:** Xi Wu, Zhipeng Yang, Jinrong Hu, Jing Peng, Peiyu He, Jiliu Zhou

**Affiliations:** ^1^Department of Computer Science, Chengdu University of Information Technology, Chengdu 610225, China; ^2^College of Electronic Engineering, Sichuan University, Chengdu 610065, China; ^3^Department of Computer Science, Xihua University, Chengdu 610039, China

## Abstract

The spatial resolution of diffusion-weighted imaging (DWI) is limited by several physical and clinical considerations, such as practical scanning times. Interpolation methods, which are widely used to enhance resolution, often result in blurred edges. Advanced superresolution scanning acquires images with specific protocols and long acquisition times. In this paper, we propose a novel single image superresolution (SR) method which introduces high-order SVD (HOSVD) to regularize the patch-based SR framework on DWI datasets. The proposed method was implemented on an adaptive basis which ensured a more accurate reconstruction of high-resolution DWI datasets. Meanwhile, the intrinsic dimensional decreasing property of HOSVD is also beneficial for reducing the computational burden. Experimental results from both synthetic and real DWI datasets demonstrate that the proposed method enhances the details in reconstructed high-resolution DWI datasets and outperforms conventional techniques such as interpolation methods and nonlocal upsampling.

## 1. Introduction

Diffusion-weighted imaging (DWI) is a noninvasive magnetic resonance modality which can be used to infer features of local tissue anatomy, composition, and microstructure from water displacement measurements [1]. Water does not diffuse equally throughout the brain and this property has been applied widely for* in vivo* analysis of white matter architecture and neuronal diseases [2, 3]. Despite the rapid development of this technology and the broad application of anisotropic diffusion properties, DWI exhibits an inherently low signal-to-noise ratio (SNR) compared with other imaging modalities. In addition, because DWI implements EPI scanning in multiple directions, the spatial resolution is relatively poor under limited physical and clinical considerations such as durable scanning time of patients.

It has been shown that the limited resolution of DWI introduces a partial volume effect (PVE) which results in bias during DWI imaging analysis [4]. The improvement of DWI spatial resolution with high SNR provides a better sensitivity for the analysis of brain structure and clinical disease [5, 6]. Moreover, high-resolution DWI could improve the estimation accuracy of diffusion tensor imaging, thus proving to be beneficial to fiber tractography and finer bundle analysis [7].

Several methods have been proposed in the literature to enhance the spatial resolution of DWI. During the acquisition stage, long acquisition times remain a primary obstacle preventing the method from being of real interest clinically. For example, Miller et al. [8] implemented five days of scanning to obtain postmortem high-resolution DWI with high SNR. In order to avoid the long scanning times, superresolution (SR) acquisition emerged as an effective technology, initially proposed for MRI, but soon adapted to DWI. Subpixel shifting in the in-plane dimension was proposed to obtain multiple low-resolution images for reconstruction into higher resolution images [9]. Anisotropic scanning was another strategy used to obtain low-resolution images for reconstruction. Scherrer et al. [10] employed a maximum of a posteriori estimation from anisotropic orthogonal acquisition to reconstruct isotropic high-resolution DWI images.

Compared with the SR acquisition implemented in specific scanning protocols, the SR algorithm in the postprocessing stage has been involved with the scene image SR reconstruction. This method category is independent of acquisition protocol and was previously implemented on MRI. The most intuitive methods for increasing resolution are interpolation methods, such as bicubic and B-spline interpolation [11]. These methods estimate the new voxels according to some smoothness assumptions which are not valid in inhomogeneous areas. Hence, interpolation methods usually result in blurred edges and artifacts in lines. Nowadays, advanced superresolution algorithms for scene images have been proposed for MRI and the main idea is reconstructing high-resolution information from the image content. Manjón et al. [12] used a nonlocal estimator to reconstruct high-resolution MRI using a single low-resolution dataset. Rousseau [13] involved multimodality MRI to improve the SR quality. Coupé et al. [7] implemented a nonlocal estimator in DWI and also incorporated *b*_0_ information to enhance the reconstruction results. Sparse representation, a recent trend in signal and image processing, has also been effectively implemented in MRI. Rueda et al. [14] reconstructed single MRI datasets based on prior knowledge with a pretrained overcomplete dictionary. Trinh et al. [15] extended the sparse representation with nonnegative expressions in order to remove noise and superresolve the two sets together.

Most of the currently used SR methods are based on the structure MRI modality and, when applied to DWI, joint information should be considered for better reconstruction [16]. Joint information comes from the redundancy acquired from adjacent scanning directions. This extra information is beneficial for enhancing the spatial resolution of DWI. Single value decomposition (SVD) plays a central role in reducing high-dimensional data to lower-dimensional data and is a classical method involved in inverse problems such as denoising [17] and restoration [18]. Recently, high-order single value decomposition (HOSVD) was used to generalize the SVD of a matrix into a high-order matrix and offered a simple yet elegant method for handling similar patches [19]. Additionally, the HOSVD basis was adapted from image content and may achieve a more sparse representation than the fixed basis. In this paper, we propose a novel SR method for DWI datasets using HOSVD. Similar to the nonlocal patch-based SR approaches successfully implemented on both MRI and DWI [7, 12, 13], HOSVD was used to construct the regularization framework in the proposed method. The merit of the HOSVD SR method stems from the adaptive HOSVD basis which results in a more accurate reconstruction. Also, the HOSVD is only implemented over similar patches in a stack, which effectively decreases the computational complexity simultaneously. This is especially useful for DWI, since the involvement of joint information from adjacent directions in DWI datasets dramatically increases the computation burden [16].

The remainder of this paper is organized as follows: we first describe the proposed method in detail and then apply it to both synthetic and* in vivo* DWI datasets for experimental evaluation. Experimental results and computational efficiency are demonstrated in Section 4 and concluding remarks are given in Section 5.

## 2. Methods

Image SR leads to an ill-posed inverse problem which is related to the LR image **y** and HR image **x**; the general model can be expressed as follows:

(1) $$\begin{array}{c} y=DHx+n, \end{array}$$

where **n** represents acquisition noise, *D* represents the decimator operator, and *H* represents the degradation function [7, 12, 13].

Based on this model, the SR image can be estimated by minimizing a least-square cost function as follows:

(2) $$\begin{array}{c} \hat{x}=arg\;\underset{x}{min}{\left\|\;y-DHx\;\right\|}^{2}. \end{array}$$

For such inverse problems, a regularization term should be added to stabilize the convergence; thus, the HR image **x** can be estimated from the LR observation **y** using the following equation:

(3) $$\begin{array}{c} \hat{x}=arg\;\underset{x}{min}\left\{\;\left\|\;y-DHx\;\right\|+\lambda R\left(\;x\;\right)\;\right\}, \end{array}$$

where *R*(**x**) is the regularization term, ‖**y** − *DH* **x**‖ is a fidelity term, and *λ* is a balancing parameter. As demonstrated by Coupé et al. [7], nonlocal patches methods can be an efficient way to define the regularization term. Instead of using a nonlocal mean estimator, we propose the implementation of a high-order SVD to be used as the estimator in this study, owing to its simple application and promising performance [19].

The HOSVD estimator clusters similar patches into a stack, in a manner similar to other patch-based methods [7, 12], and then performs an HOSVD transformation to obtain the HOSVD basis and coefficients. After the truncation of the coefficients, the patches are then reconstructed by an inverse HOSVD transform.

With this in mind, the regularization term for the superresolution process in (3) can be defined as follows:

(4) $$\begin{array}{c} R\left(\;x\;\right)=\underset{i}{\sum }\left\|\;x\left(\;i\;\right)-\psi_{HOSVD}\left(\;x\left(\;i\;\right)\;\right)\;\right\|, \end{array}$$

where *ψ*_HOSVD_ is the HOSVD base estimator.

Given an *n* × *n* patch **P**_*i*_ centered in *i*, we define *K* such similar patches (including **P**_*i*_) as {**P**_*n*_}, where 1 < *n* < *K*, and the *K* − 1 similar patches are obtained as follows.

Let us denote {**P**_*n*_} as the stack **Z** ∈ **L** ^*n*×*n*×*K*^; the HOSVD of the stack can then be defined as [20]

(5) $$\begin{array}{c} L=S\;\times_{1}\;U^{\left(1\right)}\;\times_{2}\;U^{\left(2\right)}\;\times_{3}\;U^{\left(3\right)}, \end{array}$$

where **S** is the set of coefficient matrices for a three-order tensor with *p* × *p* × *K*, ×_*j*_ stands for the *j*th mode tensor product defined in [20], and **U** ^(1)^ ∈ **L** ^*n*×*n*^, **U** ^(2)^ ∈ **L** ^*n*×*n*^, and **U** ^(3)^ ∈ **L** ^*K*×*K*^ are orthonormal unitary matrices.

After applying the HOSVD transform, the patches can be estimated by nullifying the coefficients under the assumption that the coefficients of the clean image have a sparse distribution. As indicated in [19], the coefficients can be truncated using hard thresholding as follows:

(6) $$\begin{array}{c} S^{\prime }=H_{\tau }\left(\;S\;\right), \end{array}$$

where *H*_*τ*_ denotes the hard threshold defined by $\tau =\sigma \sqrt{2\log\left(p^{2}K\right)}$ for the stack with *K* patches of size *n* × *n*. As noted in [20], the coefficients in tensor** S** are not necessarily positive and the hard thresholding is defined as the absolute value of the coefficient array:

(7) $$\begin{array}{c} H_{\tau }\left(\;S\;\right)=\left\{\begin{array}{cc} S_{i} & if\;\;abs\left(S_{i}\right)\geq \tau \\ 0 & if\;\;abs\left(S_{i}\right)\leq \tau , \end{array}\right. \end{array}$$

where **S**_*i*_ denotes the *i*th element of tensor **S**.

After truncation, the stack **Z** is reconstructed using an inverting transform with truncated coefficients to obtain the final HOSVD estimator *ψ*_HOSVD_:

(8) $$\begin{array}{c} \psi_{HOSVD}=S^{\prime }\;\times_{1}\;U^{\left(1\right)}\;\times_{2}\;U^{\left(2\right)}\;\times_{3}\;U^{\left(3\right)}. \end{array}$$

Since the DWI datasets are three-dimensional, the above method should be extended to include fourth-order HOSVD transforms for stacks with 3D similarity patches. Besides this, the threshold should be modified as $\tau =\sigma \sqrt{2\log\left(p^{3}K\right)}$ for a stack of size *n* × *n* × *n* × *K*.

In [12], a mean consistency correction followed the estimator to ensure coherence with the physical acquisition model. This was implemented in the fidelity term:

(9) $$\begin{array}{c} Y\left(\;i\;\right)-\frac{1}{L}\overset{L}{\underset{i=1}{\sum }}\hat{X}\left(\;i\;\right)=0,\;\forall p\in Y. \end{array}$$

This was done for the entirety of location *p* in the LR dataset while subsampling consistency was imposed on the reconstructed patches. Finally, the iteration process is summarized by (8) and (9) and is applied until convergence:

(10) x^t+1i=ψHOSVDxti,x^t+1=x^t+1−NNDHx^t+1−y,

where NN is the nearest neighbor interpolation and *t* is the iteration number.

In order to further improve the SR performance, the proposed HOSVD SR method can be augmented using joint information from adjacent directions in the DWI dataset [16]. For each patch **P**_*i*_, the corresponding stack **Z** was constructed with *K* similar patches which were determined as follows: the distance threshold selected all patches for which ‖**P**_*i*_ − **P**_*n*_‖ < *τ*_*d*_ was chosen as *τ*_*d*_ = 3*σ* ^2^ *n* ^2^, where *σ* is the variance of the noise. This threshold is balanced between the estimation accuracy and the computational speed as indicated in [19]. The joint information was introduced by enlarging the search window into the *M* adjacent DWI datasets, where *M* is defined as *M* = 2*m* + 1 and *m* denotes the *m* directions before and after it. In this paper, the HOSVD superresolution method which uses joint information in multiple directions is referred to as HOSVD-M.

## 3. Experiments

In order to quantitatively evaluate the quality of the reconstruction, B-spline interpolation, which has been introduced for DWI resolution enhancement in the literature [21, 22], is used for comparison. In addition to this, a nonlocal approach for image SR [12] is also involved as an effective nonlocal patch-based SR method for comparison purposes. In this section, both synthetic and* in vivo* datasets were implemented for evaluation. The patches size *n* was empirically set to 5 as suggested in [23], which was for denoising purposes primarily and also demonstrated robust results in this work. The balance parameter *λ* was set to 0.01 in all experiments. Since a sensitivity analysis for this parameter showed that the values between 0.001 and 0.2 only generated less than 0.1 dB variations of the PSNR, this means that the reconstruction has little dependence of this parameter which was also observed in the literature [14].

**Figure 1:**
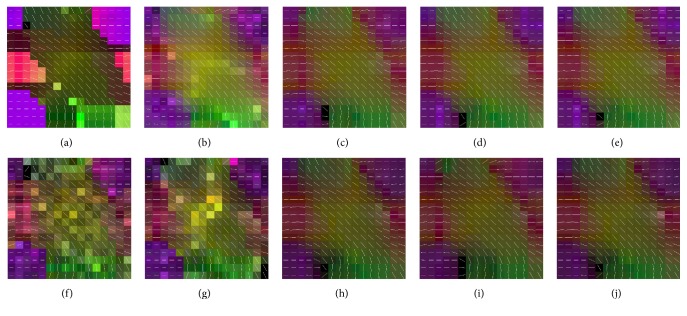
Principle eigenvectors for the tensor model applied to a synthetic phantom. (a) The original dataset, the phantom datasets reconstructed using (b) B-splines, (c) the nonlocal method, (d) the proposed HOSVD, and (e) the proposed HOSVD-M. (f) The noisy phantom (SNR = 30) and the phantom dataset reconstructed using (g) B-splines, (h) the nonlocal method, (i) the proposed HOSVD, and (j) the proposed HOSVD-M.

The simulated dataset without noise was chosen as ground truth, which consists of the 3D structure field presented at the 2012 HARDI Reconstruction Challenge [24] and occupies a 16 × 16 × 5 volume, mimicking a realistic 3D tract configuration. As shown in Figure 1(a), this dataset is comprised of five different fiber bundles which give rise to the nonplanar configurations of bending, crossing, and kissing tracts. All fiber tracts were characterized with a fractional anisotropy between 0.75 and 0.90. To better explore the proposed method, this synthetic dataset was also corrupted by Rician noise (SNR = 30) as demonstrated in Figure 1(e). Both the original dataset and the noisy set were downsampled by factor 2 using nearest neighbor interpolation along each axis. Afterwards, the LR datasets were superresolved using the B-spline method, the nonlocal method, and the proposed method, respectively. In addition to the visual comparison demonstrated in Figure 1, the angular accuracy was also measured for quantitative evaluation purposes [24]. The angular accuracy in the orientation of the estimated fiber compartments was assessed by medians of the average error (in degrees) between the estimated fiber direction and the true direction present in each voxel:

(11) $$\begin{array}{c} \bar{\theta }=\frac{180}{\pi }\arccos\left(\;\left|\;d_{true}\cdot d_{estimated}\;\right|\;\right), \end{array}$$

where the unitary vectors **d**_true_ and **d**_estimated_ are a true fiber population in the voxel and the closest of the estimated directions. For further analysis, the average error in all voxels was calculated and demonstrated in box-and-whisker diagrams. The upper and lower edges of the boxes are the 75 and 25 percentile, respectively; the smallest and biggest observations are the two ends of the whisker. The mean and median are demonstrated using red dot and line, and for each reconstruction dataset, 2% of the worst results were selected as outliers to eliminate the anomaly results.

The* in vivo* DWI dataset was acquired using a 7T Philips Achieva whole body scanner (Philips Healthcare, Cleveland, OH) equipped with a volume head coil for transmission and 32 channels. A DW dual spin-echo, SENSE accelerated msh-EPI was used to acquire the DWI data (*b*-value: 700 s/mm^2^; 15 diffusion directions), FOV = 210 × 30 × 21 mm^3^, matrix size = 300 × 300 with 15 slices, and a spatial resolution of 0.7 × 0.7 × 2 mm^3^. In order to validate the proposed approach both quantitatively and qualitatively, a gold standard image was constructed based on the* in vivo* HR DWI dataset. This was calculated by averaging 10 acquisitions of high-resolution DW images in the image space (0.7 × 0.7 × 2 mm^3^). LR images were then used in the experiment and were simulated by downsampling the gold standard by factor 2 using nearest neighbor interpolation along each axis (i.e., $\left[\begin{array}{ccc} 2 & 2 & 2 \end{array}\right]$), which resulted in simulated LR images of size 1.4 × 1.4 × 4 mm^3^.

In order to quantitatively evaluate the superresolved DWI dataset, two objective measurement matrices, namely, the peak signal-to-noise ratio (PSNR) and structural similarity (SSIM) [25], were used. The PSNR measures the extent to which noise has been suppressed and SSIM measures the structural and perceptual similarities between the original and reconstructed images:

(12) $$\begin{array}{c} SSIM\left(\;x,y\;\right)=\frac{\left(2\mu_{x}\mu_{y}\right)\left(2\sigma_{xy}+c_{2}\right)}{\left(\mu_{x}^{2}+\mu_{y}^{2}+c_{1}\right)+\left(\sigma_{x}^{2}+\sigma_{y}^{2}+c_{2}\right)}, \end{array}$$

where *μ*_*x*_ and *μ*_*y*_ are the mean values of images *x* and *y*. The terms *σ*_*x*_ and *σ*_*y*_ are the standard deviation of *x* and *y*, respectively, *σ*_*xy*_ is the covariance between them, and the constants *c*_1_ and *c*_2_ are chosen as suggested [25].

**Figure 2:**
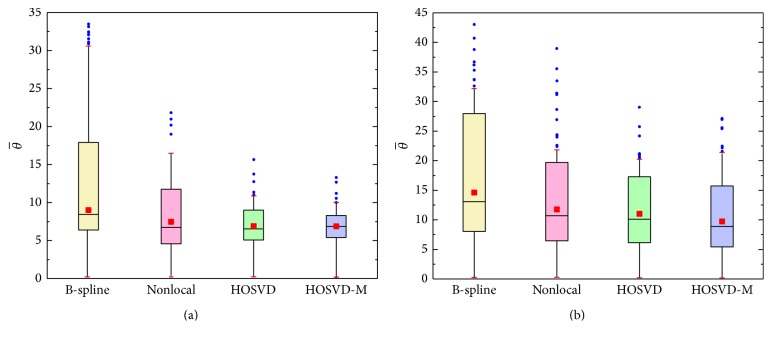
The box-and-whisker diagrams of the distribution of the angular accuracy $\bar{\theta }$. In each box, the edges of the box represent the 25th and 75th percentiles, while the mean and median are reported as red dot and line, respectively. The whiskers extend to the smallest and largest observation in the data, with the 2% of the worst results considered as outliers individually plotted as blue dots.

Tensor estimation of the* in vivo* DWI dataset was evaluated quantitatively between the superresolved dataset and the gold standard. First, the diffusion tensor field and principal eigenvectors were computed using CAMINO [26] and are demonstrated in Figure 1; Figure 2 contains the mean angular error estimated by (11). The fractional anisotropy (FA) map and colormap of the estimated DTI were calculated and are shown for comparison. Finally, the primary direction of the tensor is also demonstrated for visual comparison.

## 4. Results

Figure 1 illustrates the principle eigenvector for the tensor model in the synthetic phantom and the results reconstructed using B-spline interpolation, nonlocal upsampling, the proposed HOSVD, and the proposed HOSVD-M. It is evident from the results in the figure that the superresolved methods dramatically outperformed the interpolation methods. The proposed method achieved the best results, by visual inspection, for both noisy and no-noise configurations. This is likely due to the adaptive HOSVD bases derived from the stacked patches, which are more suitable for reconstruction. Quantitative comparisons were provided in Figure 2 for more comprehensive evaluation. The expected inability of reconstruction using interpolation method was clearly reflected as the highest $\bar{\theta }$ value in both original and noisy phantom datasets. Meanwhile, the proposed HOSVD and HOSVD method achieved the best reconstruction quality with the lowest $\bar{\theta }$ value and remarkable stability. It can be seen in Figure 2(a), in the original dataset, the proposed two HOSVD methods achieved narrower angle error distribution compared with other methods. Besides this, in the noisy dataset (Figure 2(b)), the proposed HOSVD-M achieved lower mean and median results compared with the NLM method. Moreover, both the HOSVD methods demonstrated plausible stability with significantly less outliers than the NLM method.

**Figure 3:**
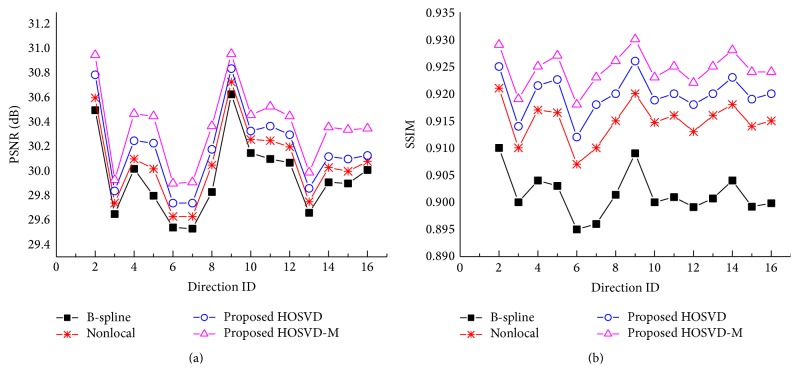
PSNR and SSIM estimation between the gold standard and images reconstructed from a simulated LR image. (a) Plots showing the PSNR for the compared methods. (b) Plots showing the SSIM for the compared methods.

**Figure 4:**
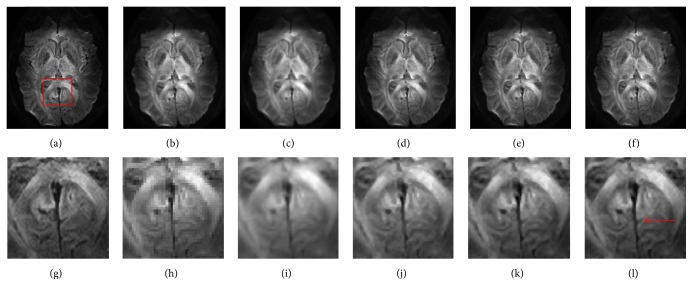
Diffusion-weighted image reconstruction tests obtained using different methods. (a) The gold standard. (b) The downsampled dataset. Results of (c) B-spline reconstruction, (d) nonlocal method, (e) the proposed HOSVD, and (f) the proposed HOSVD-M. Enlarged details of the (g) gold standard, (h) downsampled dataset, (i) B-spline reconstruction, (j) nonlocal method, (k) the proposed HOSVD, and (l) the proposed HOSVD-M.

The reconstructed results for* in vivo* DWI data are demonstrated both quantitatively and qualitatively in Figures 3 and 4, respectively.  Figure 3 displays the PSNR and SSIM for the reconstructed DWI dataset using the above methods. As indicated previously, the patch-based SR methods obviously outperformed the interpolation methods. The proposed HOSVD methods outperformed the nonlocal upsampling method in most of the diffusion directions and the HOSVD-M method achieved the best results in most of them.  Figure 4 shows a visual comparison of the reconstructed DWI images. The interpolated results (Figure 4(b)) were the blurriest. Images reconstructed using the proposed method were the most similar to the original images. The enlarged region (Figure 4(l)) demonstrates that the proposed HOSVD clearly reconstructed the spatial features of the cracked area as indicated in the red arrow, as compared with the same area reconstructed by other methods, in which the edges are blurry and difficult to distinguish.

**Figure 5:**
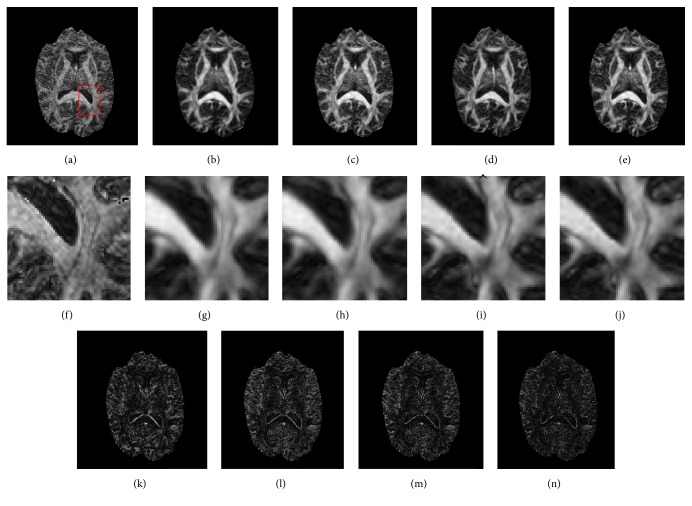
FA maps estimated using the gold standard and several other methods. (a) FA maps estimated using the gold standard; FA maps obtained for the reconstructed dataset using the (b) B-spline, (c) nonlocal method, (d) the proposed HOSVD, and (e) the proposed HOSVD-M. Enlarged details of the (f) golden standard, (g) B-spline reconstruction, (h) nonlocal method, (i) the proposed HOSVD, and (j) the proposed HOSVD-M. The red ROIs indicate a detailed reconstruction. Visually, the FA map obtained using the proposed method is closer to the FA of the gold standard. FA residual of the (k) B-spline, (l) nonlocal method, (m) the proposed HOSVD, and (n) the proposed HOSVD-M.

**Figure 6:**
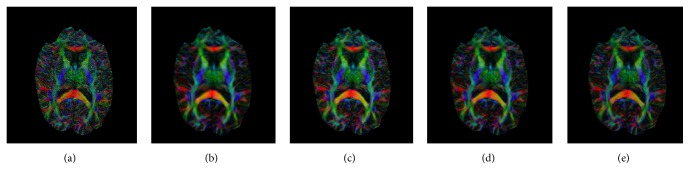
(a) FA colormap for the gold standard; FA colormaps for the reconstructed dataset using (b) B-spline, (c) nonlocal method, (d) the proposed HOSVD, and (e) the proposed HOSVD-M.

**Figure 7:**
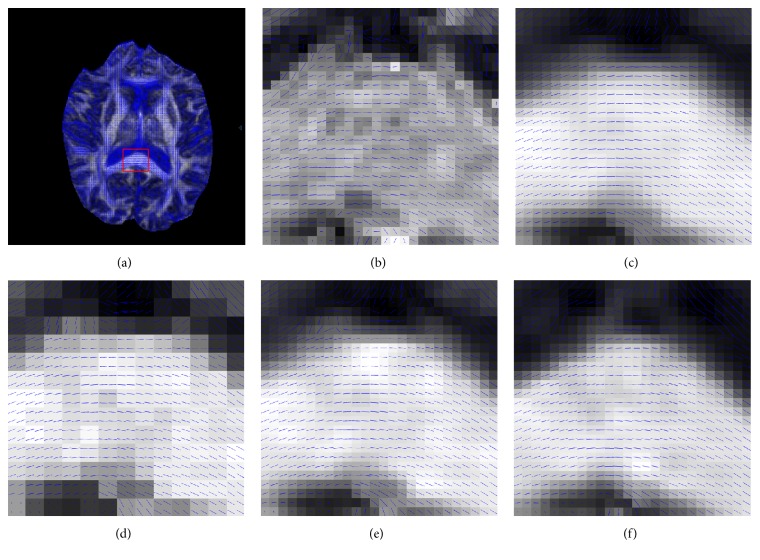
Diffusion tensor estimations on a central slice, centered and zoomed on the corpus callosum. (a) Tensors estimated on the gold standard; the red ROIs indicate a detailed reconstruction. Tensors estimated on the reconstructed dataset using (b) B-spline, the (c) nonlocal method, (d) the proposed HOSVD, and (e) the proposed HOSVD-M. The blue stick indicates the main eigenvector of the diffusion tensor.

Figures 5 and 6 demonstrate the tensor estimation results using the superresolved DWI datasets.  Figure 5 shows the FA map for the estimated DTI datasets. The proposed HOSVD-M method achieved the best results in the enlarged areas and retained most of the structure and tissue from the original images. It can be seen more clearly that, in the residual map, the result of HOSVD-M remained less in structure information compared with others. The fiber direction indicated in the FA colormap is shown in Figure 6. It can be observed in Figure 6(e) that the proposed HOSVD-M obtained robust direction reconstruction results. For example, in the corpus callosum bundle, the color of most voxels remained the same. This coincides with Figure 7(f) in which the primary eigenvectors for the voxels in the corpus callosum maintain the same direction.

**Figure 8:**
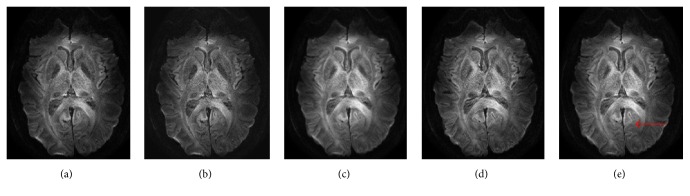
Diffusion-weighted image reconstruction from* in vivo* golden standard dataset. The resolution was thus increased from 0.7 × 0.7 × 2 mm^3^ to 0.35 × 0.35 × 1 mm^3^. (a) The gold standard. Results of (b) B-spline reconstruction, (c) nonlocal method, (d) the proposed HOSVD, and (e) the proposed HOSVD-M.

In addition, we applied our method directly to the* in vivo* golden standard DWI dataset.  Figure 8 shows the results of the reconstruction using different method. As it can be observed, the proposed method achieved plausible reconstruction as well. As pointed out by red arrow, the HOSVD-M preserved the fine detail and reconstructed explicit crack boundary which may be beneficial for further applications.

## 5. Discussion and Conclusion

In this work, we investigated a novel patch-based single image superresolution method to increase the spatial resolution of DWI datasets. The proposed method introduced HOSVD into the SR framework as a regularization term to achieve better image reconstruction and more efficient computation. Joint information from adjacent DWI directions was also involved to make further improvements. Both synthetic and* in vivo* DWI datasets were implemented for evaluation of the DWI reconstruction and DTI estimation.

Compared with conventional interpolation and patch-based SR methods, the improvements made by the proposed HOSVD method can be contributed to two features. The first is adapted HOSVD basis acquired from a stack of similar patches. This technique obtained bases adaptively, according to image content, and achieved a more effective reconstruction result. The second feature is the introduction of joint information from adjacent directions in the DWI datasets. As noted in [16], the adjacent directions contain a significant amount of image redundancy and the encapsulation of both the processed and adjacent directions effectively benefits the reconstruction.

Computational complexity is another important issue for patch-based methods as well as DWI processing. All experiments were performed on a PC running MATLAB R2013b in Windows 7, with an Intel(R) core i7-4600U processor and 8 GB of RAM. For a typical DWI dataset with a matrix size of 128 × 128, 60 slices, and 32 directions, the runtime for a single direction was approximately 8 minutes for nonlocal upsampling, 3 minutes for the proposed HOSVD, and 5 minutes for the proposed HOSVD-M. This increase in speed is likely due to an inherent dimensional decreasing property of the SVD as well as HOSVD. Specifically, compared with nonlocal methods which average every patch to estimate the reconstructed patches, the proposed method only manipulates a portion of patches with high similarity. This induced a faster convergence speed. We also expect that the implementation of parallel computing on graphic processing units could further speed up the reconstruction. This will require further research in future studies.

In this paper, we proposed a patch-based single image superresolution method which involved applying a high-order SVD to a DWI dataset. The adaptive HOSVD bases acquired from the image ensured a more accurate image reconstruction and manipulation of similar patch stacks led to a reduction in computational complexity. Quantitative and qualitative comparisons of the traditional interpolation and nonlocal patch-based methods demonstrate the competitive results obtained for both DWI reconstruction and DTI estimation.
